# Deep learning for automated detection of neovascular leakage on ultra-widefield fluorescein angiography in diabetic retinopathy

**DOI:** 10.1038/s41598-023-36327-6

**Published:** 2023-06-06

**Authors:** Peter Y. Zhao, Nikhil Bommakanti, Gina Yu, Michael T. Aaberg, Tapan P. Patel, Yannis M. Paulus

**Affiliations:** Department of Ophthalmology and Visual Sciences, W.K. Kellogg Eye Center, University of Michigan, 1000 Wall Street, Ann Arbor, MI 48105 USA

**Keywords:** Diagnostic markers, Retinal diseases, Diabetes complications

## Abstract

Diabetic retinopathy is a leading cause of blindness in working-age adults worldwide. Neovascular leakage on fluorescein angiography indicates progression to the proliferative stage of diabetic retinopathy, which is an important distinction that requires timely ophthalmic intervention with laser or intravitreal injection treatment to reduce the risk of severe, permanent vision loss. In this study, we developed a deep learning algorithm to detect neovascular leakage on ultra-widefield fluorescein angiography images obtained from patients with diabetic retinopathy. The algorithm, an ensemble of three convolutional neural networks, was able to accurately classify neovascular leakage and distinguish this disease marker from other angiographic disease features. With additional real-world validation and testing, our algorithm could facilitate identification of neovascular leakage in the clinical setting, allowing timely intervention to reduce the burden of blinding diabetic eye disease.

## Introduction

Diabetic retinopathy (DR) is a leading cause of vision loss worldwide, often affecting working-age individuals^1^. It is the most common microvascular complication of diabetes mellitus (DM), affecting nearly one-third of those with DM^2^. In the early non-proliferative stages of DR (NPDR), regular screening is recommended to identify individuals at risk of progression to proliferative DR (PDR)^3^. If PDR develops, early detection and treatment with laser and/or intravitreal anti-VEGF injections reduces the risk of permanent vision-threatening complications^4,5^. It is therefore imperative to accurately identify patients with PDR to guide timely treatment.

Eye exams and color fundus photographs are the standard of care to detect the presence of neovascularization (NV), the defining feature of PDR. NV must be discerned from other abnormal vascular features in NPDR, such as microaneurysms, dot-blot hemorrhages, venous beading, and intraretinal microvascular abnormalities^6^. Another imaging modality that complements eye exams is fluorescein angiography (FA). FA uses the fluorescent properties of fluorescein dye to highlight abnormal vascular features that may be more difficult to observe with an eye exam alone^7^. For example, the NV lesions that are diagnostic of PDR cause leakage on FA, which is characterized by bright hyperfluorescence that progressively enlarges over time with hazy borders. However, leakage caused by NV must still be distinguished from other angiographic findings in DR. For example, microaneurysms may appear as bright hyperfluorescent foci, retinal edema may appear as areas of late hyperfluorescence, and vascular incompetence manifests as late hyperfluorescence along retinal vessels. Thus, the interpretation of FAs may provide additional diagnostic value in DR beyond examination and color fundus photographs, but also requires a trained clinician to identify NV and PDR.

Convolutional neural networks (CNNs) have been used in the automated classification of eye diseases including age-related macular degeneration, retinopathy of prematurity, and glaucoma^8–10^. CNNs have also been used in the detection of referral-warranted diabetic retinopathy from color fundus photographs^11,12^. In this study, we trained a deep learning algorithm to detect leakage from retinal NV on ultra-widefield fluorescein angiography (UWF-FA), an imaging modality that provides a 200-degree field of view for FA. Retinal NV defines PDR, and accurate detection of NV facilitates early treatment before progression to vision-threatening complications. Our algorithm was able to accurately classify neovascular leakage in the presence of other hyperfluorescent and hypofluorescent lesions on UWF-FA images. With further improvements in performance, adoption of such an algorithm could allow for more consistent identification of NV, facilitating early treatment of patients with PDR and reducing the burden of blinding diabetic eye disease.

## Results

### Patient and imaging characteristics

The study included 678 images from 377 patients. Table 1 shows the demographic and clinical characteristics of the 377 patients.

**Table 1:** Patient baseline characteristics.

Total N = 377	n	%
Sex		
Male	209	55
Female	168	45
Race		
White	249	66
Black	90	24
Asian	16	4
Other	22	6
Type 1 DM	69	18
Type 2 DM	308	82

	Mean	SD
Age	58.6	13.4
Last systolic BP	136.8	22.4
Hemoglobin A1c (%)	8.0	2.0
BMI	32.6	8.7

There was a slightly greater proportion of male patients (55%; n = 209) and most were White (66%; n = 249) with a substantial minority who were Black (24%; n = 90). Mean age was 58.6 years (SD 13.4), mean BMI was 32.6 kg/m^2^ (SD 8.7), and mean systolic blood pressure was 136.8 mmHg (SD 22.4). Most patients had type 2 DM (82%; n = 308), and mean hemoglobin A1c was 8.0% (SD 2.0). Eye-level characteristics of the 678 images are shown in Table 2.

**Table 2:** Eye characteristics.

N = 678	Mean	SD
LogMAR VA	0.36	0.36
Snellen VA Equivalent	20/46	

	n	%
Eye laterality		
OD	366	54
Lens status		
Phakic	237	35
Pseudophakic	425	63
Aphakic	16	2
DR severity		
No DR	70	10
Mild NPDR	102	15
Moderate NPDR	135	20
Severe NPDR	90	13
PDR	163	24
History of macular edema	290	43
Neovascular leakage on FA	120	18

Eyes with varying clinical DR severity ranging from no DR to PDR were included, with DR severity determined by eye exam with the treating retinal specialist. Mean logMAR visual acuity was 0.36 (SD 0.36), or Snellen equivalent 20/46. Severity of DR was determined on clinical exam and evaluation by a retinal specialist. 10% (n = 70) of eyes had no DR, 15% (n = 102) of eyes had mild NPDR, 20% (n = 135) of eyes had moderate NPDR, and 13% (n = 90) of eyes had severe NPDR. 24% (n = 163) of eyes had PDR. A substantial proportion of eyes had a history of macular edema (43%; n = 290). Graders identified the presence of angiographic neovascular leakage in 18% (n = 120) of images. Most eyes were pseudophakic (63%; n = 425).

### Algorithm performance

**Figure 1:**
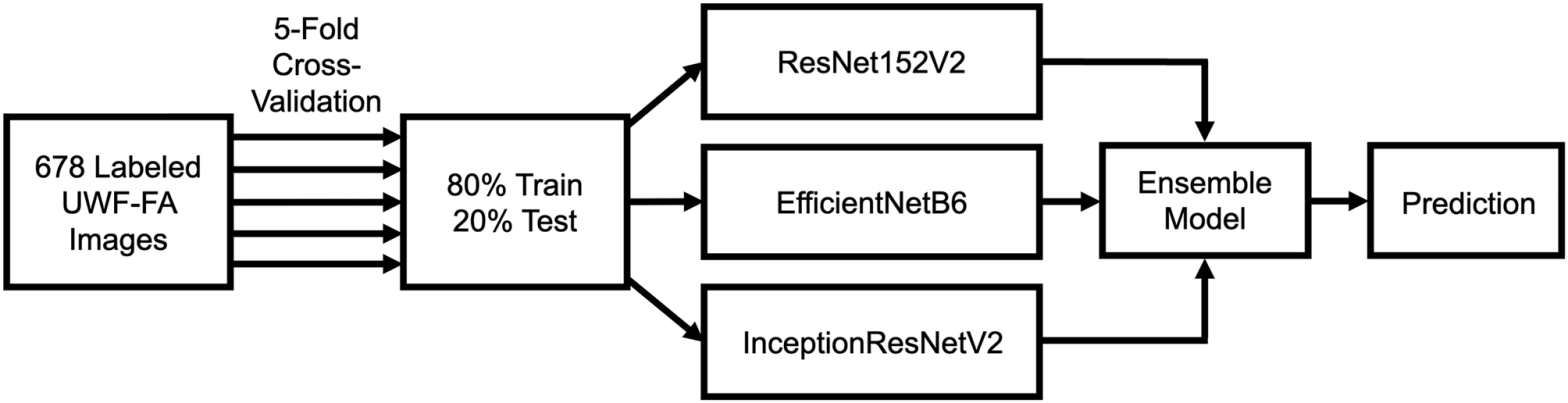
Architecture of the deep learning classifier. Five-fold cross-validation was used with an 80%/20% train/test split. Predictions from the ResNet152V2, EfficientNetB6, and InceptionResNetV2 convolutional neural networks were ensembled using model averaging.

We trained three CNNs and evaluated performance of the model-averaged ensemble classifier through five-fold cross-validation (Fig. 1).

The component CNNs were selected based on performance in a variety of prior ophthalmic applications^13–19^. Since the data set of 678 images contained only 120 images (18%) with grader-identified neovascular leakage, additional weight was placed on this classification to address the class imbalance.

**Figure 2:**
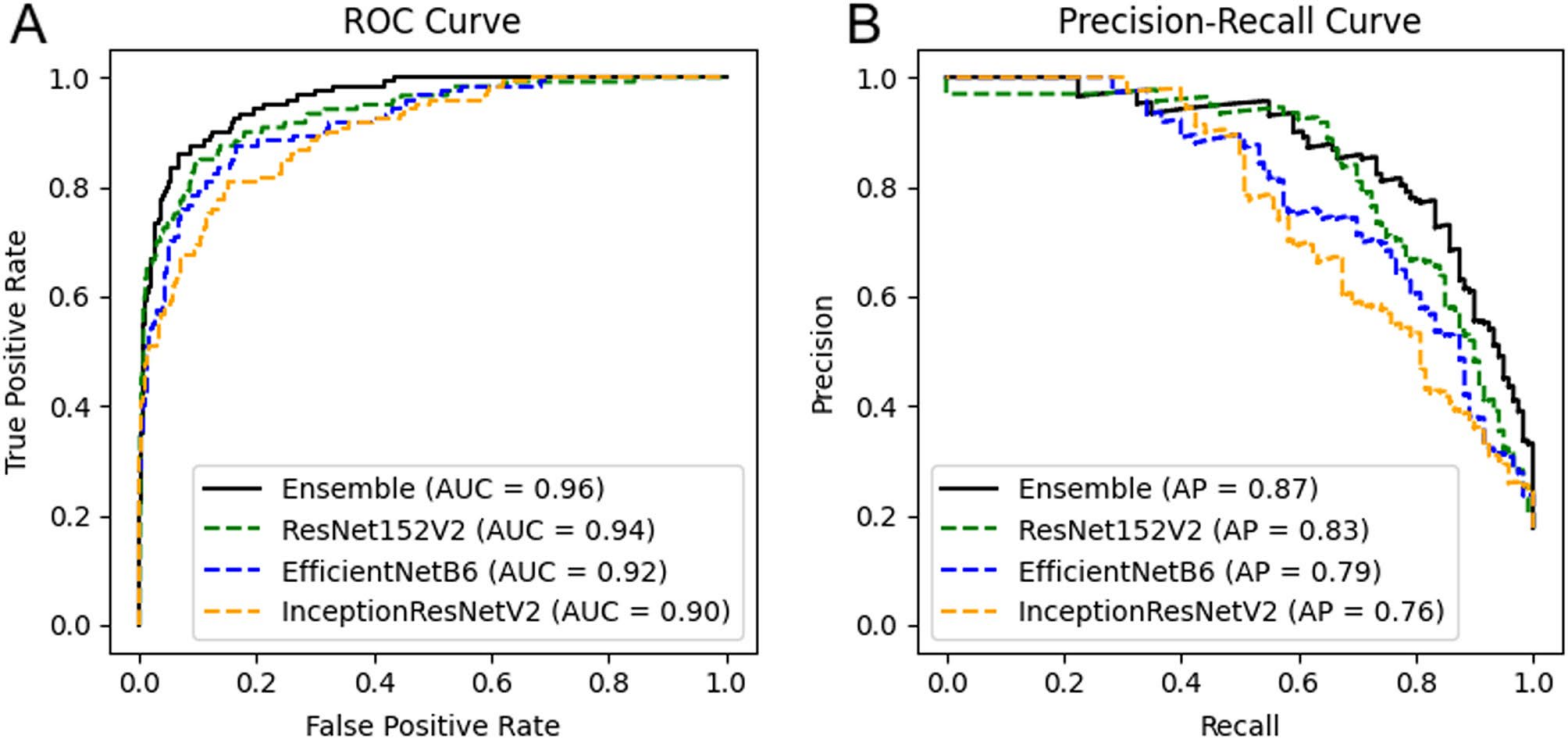
Receiver operating characteristic and precision-recall curves for detection of leakage from neovascularization by the deep learning classifier.

Figure 2A shows the receiver operating characteristic (ROC) curves obtained from five-fold cross-validation. Area under the ROC curve (AUC) was 0.96 for the model-averaged ensemble predictor. The AUCs for each individual CNN in the ensemble were 0.90 for InceptionResNetV2, 0.92 for EfficientNetB6, and 0.94 for ResNet152V2. Figure 2B shows the precision-recall (PR) curves from five-fold cross-validation. The average precision was 0.87 for the ensemble predictor. Individual CNN average precisions were 0.76 for InceptionResNetV2, 0.79 for EfficientNetB6, and 0.83 for ResNet152V2. Table 3 lists the metrics obtained within each fold of training and testing for the ensembled predictor. At the selected operating point, sensitivity was 0.82, specificity was 0.95, and precision was 0.77.

**Table 3:** Cross-validation metrics.

	Fold 1	Fold 2	Fold 3	Fold 4	Fold 5	Overall
Area under ROC curve	0.95	0.97	0.98	0.94	0.98	0.96
Average precision	0.84	0.92	0.93	0.83	0.92	0.87
Accuracy	0.90	0.95	0.94	0.90	0.94	0.92
Sensitivity	0.74	0.75	0.92	0.79	0.90	0.82
Specificity	0.93	0.99	0.95	0.93	0.95	0.95
Precision	0.68	0.95	0.79	0.73	0.76	0.77

**Figure 3:**
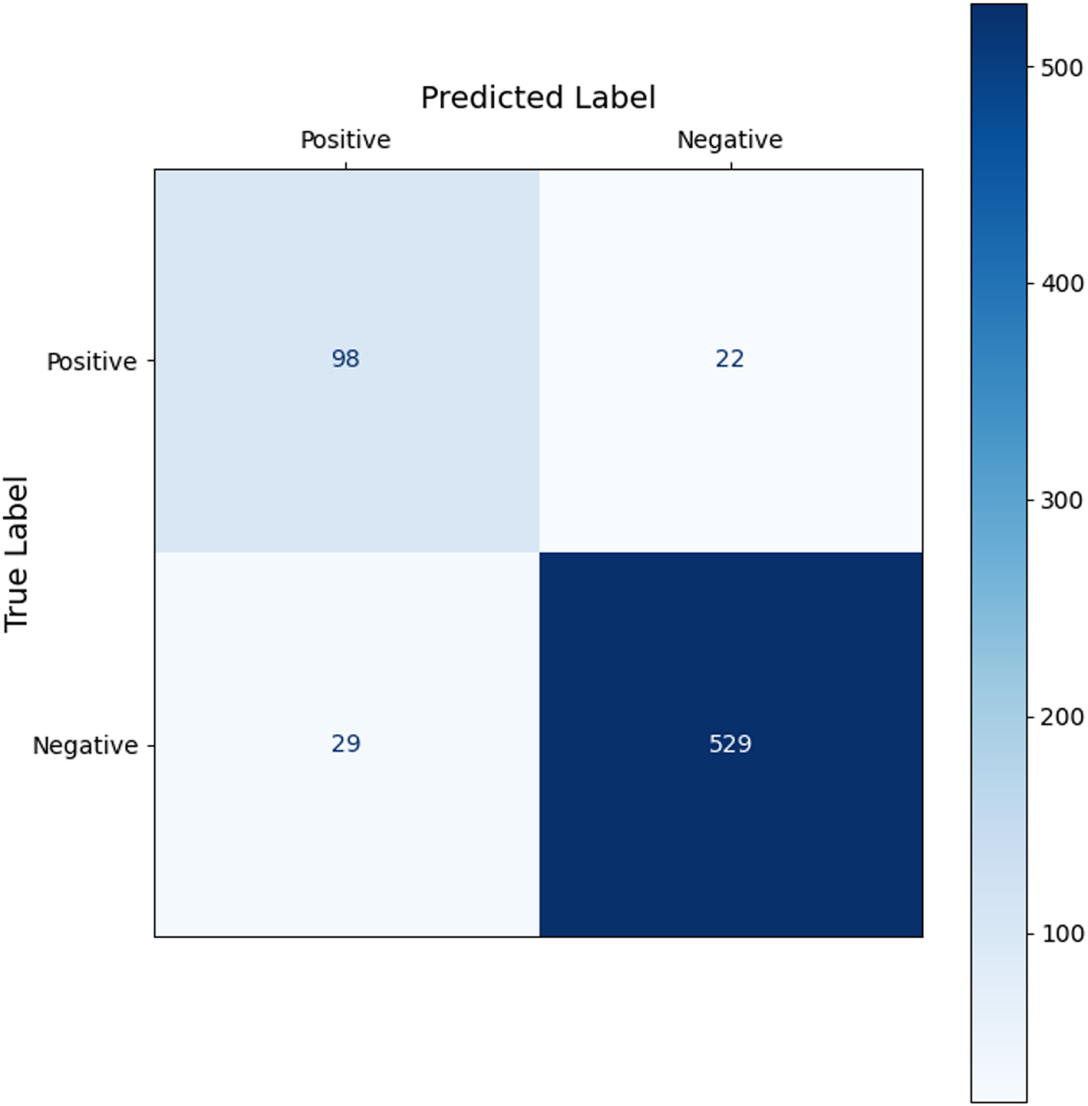
Confusion matrix comparing grader labels and algorithm predictions. Most of the fluorescein angiogram images were negative for neovascularization.

Figure 3 shows the confusion matrix for the ensemble classifier at the selected operating point. Most of the positive and negative images were corrected identified by the model, but a proportion of images were false-positives or false-negatives.

Supplementary Figure 1 shows images of the false-positive and false-negative algorithm predictions from Fold 1 of cross-validation. Some potential reasons for false-negative predictions in these images (Supplementary Fig. 1) included NV that exhibited slow leakage of fluorescein, very small foci of NV, NV near the optic disc, and concurrent presence of other causes of leakage. Other causes of leakage observed included staining around retinal venules and retinal edema. Potential reasons for false-positive predictions in these images (Supplementary Fig. 2) included window defect from fibrosis or scar, aberrant non-neovascular vessels (such as intraretinal microvascular abnormalities or IRMA), and bright fluorescence from other causes of leakage such as clusters of microaneurysms. Forty randomly-selected images and corresponding saliency maps are included in Supplementary Fig. 3 (true-positive predictions), Supplementary Fig. 4 (true-negative predictions), Supplementary Fig. 5 (false-positive predictions), and Supplementary Fig. 6 (false-negative predictions).

While no standardized external test set exists for UWF-FA images in diabetic retinopathy, to conduct a pilot external validation study we used UWF-FA images of diabetic retinopathy found in the American Society of Retinal Specialists (ASRS) Image Bank. The algorithm correctly classified 17 out of 17 (100%) images as true-positive for NV leakage, and 4 out of 5 (80%) images as true-negative for NV leakage. 1 out of 5 (20%) images was classified as false positive, and there were no false negatives.

## Discussion

Our deep learning algorithm was able to detect neovascular leakage in UWF-FA images containing other DR disease features that cause hyper- and hypo-fluorescent angiographic changes. To our knowledge, an algorithm to perform this classification task has not previously been constructed. Our image data set was obtained from a well-characterized group of patients with DM and varying stages of DR^20^. The algorithm may be useful in augmenting ophthalmologists’ or retinal specialists’ ability to discern neovascular leakage on fluorescein angiography in the clinical setting.

Deep learning algorithms have been created for screening, classification, and segmentation of numerous eye conditions^8,9,11,21,22^. In diabetic retinopathy, deep learning has been used to screen patients with diabetes mellitus (DM) for referral-warranted diabetic retinopathy (DR)^11,23^, and to identify the severity of DR^24^. For example, Dai et al. achieved an AUC of 0.97 for detection of PDR with a ResNet-based classifier^24^. Ting et al. achieved an AUC of 0.96 for detection of vision-threatening DR used a VGGNet-based classifier^25^. However, many studies including these used 50-degree color fundus photographs. Peripheral vascular lesions not visible on traditional photography are commonly found in DR and may be important prognostic indicators^26,27^. Our study used UWF imaging with a 200-degree field of view to include NV lesions outside of the posterior pole^28^. A study by Nagasawa et al*.* used non-FA UWF imaging to detect treatment-naïve PDR with an AUC of 0.97^29^. However, in their study the classifier only had to distinguish between images of normal subjects and images of subjects with PDR, whereas discriminating between NPDR and PDR may be a more challenging task. Our algorithm trained on UWF-FA images achieved an AUC of 0.96 to detect NV leakage, a finding that is diagnostic of PDR. Sickle cell retinopathy is another retinal vascular disease which may also progress into a proliferative stage characterized by neovascularization and tractional retinal detachment. Cai et al. trained an InceptionV4 network to detect seafan neovascularization from ultra-wide-field fundus photographs, achieving sensitivity and specificity of 0.97^30^. Early detection of proliferative vascular disease using automated methods may facilitate early treatment to reduce the risk of vision loss.

Our data included 163 eyes classified as PDR by a clinician, whereas only 120 images were labeled by graders as positive for NV leakage. This discrepancy could be explained by clinical scenarios in which a clinician would diagnose PDR despite lack of evidence of NV on UWF-FA. These could include development of vitreous hemorrhage in an eye with known pre-existing diabetic retinopathy, imaging of a patient who had intravitreal injection performed for proliferative disease preceding referral (causing regression of NV on imaging), neovascularization in the far periphery visible clinically but not visualized on UWF-FA, or iris neovascularization. False-positive predictions by the algorithm were not statistically different (Chi-Squared Test) between eyes with PDR without NV leakage on imaging, and eyes with NPDR without NV leakage on imaging.

Fluorescein angiogram results are typically recorded as a collection of a dozen or more image frames reflecting the different time points and phases of the angiogram. A potential use of the algorithm would be identifying image frames in which neovascularization is detected, indicating the most important frames to review. In this role, the algorithm would facilitate clinical diagnosis. Deep learning has been used to detect abnormalities on FA in other retinal diseases such as retinopathy of prematurity and age-related macular degeneration^31–33^. Since PDR is a cause of vision-threatening complications of diabetes mellitus, early diagnosis is key to obtaining appropriate treatment to prevent vision loss^4,5^. Leakage from NV is present in active, untreated proliferative diabetic retinopathy, and often resolves with treatment of PDR.

The limitations of this study include the lack of a larger, standardized external test image set, the inherent limitations of a retrospective, single-center study, inability to assess for diabetic macular edema, and that images used were only from the early venous phase of fluorescein angiography. No standardized external test image set exists for this classifier. We used a data set of 22 images from the ASRS Image Bank as a pilot data set for external validation, but a larger, standardized external test set would be needed to confirm the algorithm’s generalizability. Improved data capture and sharing standards through the National Eye Institute Bridge2AI initiative or a model-to-data approach could be used to confirm external validity^34^. Although diabetic macular edema does cause leakage on FA, the preferred imaging modality for detection of diabetic macular edema is optical coherence tomography and not FA. A multi-modality imaging approach with optical coherence tomography would be needed to incorporate detection of diabetic macular edema which is an additional vision-threatening complication of DM. Finally, we chose the early venous phase of FA because leakage from NV appeared to be most prominent during this phase in contrast to other angiographic findings. We reasoned that earlier phases of FA would be less likely to exhibit leakage from neovascularization, and that in the later phases of FA, hyperfluorescence from staining would be more difficult to distinguish from leakage. However, leakage from NV may also be present during other phases, and the temporal pattern of leakage could provide additional information to the model. Analysis of videos of FA could also be of benefit, but video FA is not widely recorded which limits its generalizability.

In summary, we trained a deep learning algorithm to detect the presence of neovascular leakage in UWF-FA images from patients with diabetic retinopathy. With additional testing to verify external validity, the algorithm could help guide early identification and treatment of proliferative diabetic retinopathy.

## Methods

We included patients 18 years of age or older with DM who received a diagnosis of diabetic retinopathy by a retina-trained clinician and had an ultra-widefield fluorescein angiography (UWF-FA) study performed between January 2009 and May 2018 at two sites of a tertiary academic medical center (Kellogg Eye Center Ann Arbor and Grand Blanc). Images were obtained retrospectively from a previously generated data set used for quantification of retinal neovascularization^20^. Briefly, the images were captured with a UWF scanning laser ophthalmoscopy device (Optos 200Tx or Optos California; Optos PLC). Images from the venous phase of the fluorescein angiogram were used because leakage from NV was most prominent during this phase compared to other angiographic findings. Images were excluded if image quality was too poor to identify distinct fundus features. Images were labeled with neovascular leakage by graders who underwent training as described previously^20^. Graders were masked to patient data. A fellowship-trained retinal specialist (P.Y.Z.) verified grader labels. Images were cropped to the central 1792 × 1280 and downsampled to 896 × 640. This study adhered to the tenets of the Declaration of Helsinki. The study was initiated after approval by the University of Michigan Institutional Review Board (HUM00120509, PI: Y.M. Paulus), which approved an exemption for the requirement to obtain informed consent.

For the deep learning algorithm, we trained three CNNs: ResNet152V2, EfficientNetB6, and InceptionResNetV2. Each network was pre-trained on ImageNet and then trained on the UWF-FA data set. The algorithm as evaluated using five-fold cross-validation, which each fold consisting of an 80% training set and 20% test set. To generate an ensembled prediction, we averaged the predictions of each of the three CNNs. The Adam algorithm was used for optimization, and the learning rate was set to 0.0005. The batch size was set to 16. Training images were randomly augmented with horizontal and/or vertical translation of up to 10%, random horizontal and vertical flip, image rotation of up to 72 degrees, and image zoom between 90 and 110%. Training was set to a maximum of 35 epochs, with early stopping if the training loss did not decrease after 8 epochs. Computation was performed using Keras with TensorFlow version 2.7.0 as backend on the University of Michigan High Performance Computing Cluster (16 GB NVIDIA Tesla V100; NVIDIA Corporation). Statistical analyses were performed using Excel and Python version 3.9.7 with scikit-learn module version 1.0.1.

## Supplementary Information

Supplementary Legends.

Supplementary Figure S1.

Supplementary Figure S2.

Supplementary Figure S3.

Supplementary Figure S4.

Supplementary Figure S5.

Supplementary Figure S6.

## Data Availability

The data that support the results of this study are not publicly available in respect of patient confidentiality. Programming code and algorithm weights are available upon reasonable request with the corresponding authors.

## References

[1] Zhang X (2010). Prevalence of diabetic retinopathy in the United States, 2005–2008. JAMA.

[2] Yau JWY (2012). Global prevalence and major risk factors of diabetic retinopathy. Diabetes Care.

[3] Flaxel CJ (2020). Diabetic retinopathy preferred practice pattern®. Ophthalmology.

[4] Early photocoagulation for diabetic retinopathy. ETDRS report number 9. Early Treatment Diabetic Retinopathy Study Research Group. *Ophthalmology.* **98**, 766–785 (1991). 2062512

[5] Writing Committee for the Diabetic Retinopathy Clinical Research Network *et al.* Panretinal photocoagulation vs intravitreous ranibizumab for proliferative diabetic retinopathy: A randomized clinical trial. *JAMA.* **314**, 2137 (2015). 10.1001/jama.2015.15217 PMC5567801 26565927

[6] Antonetti DA, Klein R, Gardner TW (2012). Diabetic retinopathy. N. Engl. J. Med..

[7] Salz DA, Witkin AJ (2015). Imaging in diabetic retinopathy. Middle East Afr. J. Ophthalmol..

[8] Yim J (2020). Predicting conversion to wet age-related macular degeneration using deep learning. Nat. Med..

[9] Brown JM (2018). Automated diagnosis of plus disease in retinopathy of prematurity using deep convolutional neural networks. JAMA Ophthalmol..

[10] Liu H (2019). Development and validation of a deep learning system to detect glaucomatous optic neuropathy using fundus photographs. JAMA Ophthalmol..

[11] Gulshan V (2016). Development and validation of a deep learning algorithm for detection of diabetic retinopathy in retinal fundus photographs. JAMA.

[12] Kim TN (2021). Comparison of automated and expert human grading of diabetic retinopathy using smartphone-based retinal photography. Eye (Lond.).

[13] Lu W (2018). Deep learning-based automated classification of multi-categorical abnormalities from optical coherence tomography images. Transl. Vis. Sci. Technol..

[14] Shibata N (2018). Development of a deep residual learning algorithm to screen for glaucoma from fundus photography. Sci. Rep..

[15] Ting DSW (2019). Artificial intelligence and deep learning in ophthalmology. Br. J. Ophthalmol..

[16] Xie Y (2020). Screening candidates for refractive surgery with corneal tomographic-based deep learning. JAMA Ophthalmol..

[17] Kuo M-T (2021). Comparisons of deep learning algorithms for diagnosing bacterial keratitis via external eye photographs. Sci. Rep..

[18] Pachade S, Porwal P, Kokare M, Giancardo L, Mériaudeau F (2021). NENet: Nested EfficientNet and adversarial learning for joint optic disc and cup segmentation. Med. Image Anal..

[19] Rasheed HA (2022). DDLSNet: A novel deep learning-based system for grading funduscopic images for glaucomatous damage. Ophthalmol. Sci..

[20] Yu G (2020). Quantification of retinal nonperfusion and neovascularization with ultrawidefield fluorescein angiography in patients with diabetes and associated characteristics of advanced disease. JAMA Ophthalmol..

[21] Asaoka R, Murata H, Iwase A, Araie M (2016). Detecting preperimetric glaucoma with standard automated perimetry using a deep learning classifier. Ophthalmology.

[22] Peng Y (2019). DeepSeeNet: A deep learning model for automated classification of patient-based age-related macular degeneration severity from color fundus photographs. Ophthalmology.

[23] Abràmoff MD, Lavin PT, Birch M, Shah N, Folk JC (2018). Pivotal trial of an autonomous AI-based diagnostic system for detection of diabetic retinopathy in primary care offices. NPJ Digit. Med..

[24] Dai L (2021). A deep learning system for detecting diabetic retinopathy across the disease spectrum. Nat. Commun..

[25] Ting DSW (2017). Development and validation of a deep learning system for diabetic retinopathy and related eye diseases using retinal images from multiethnic populations with diabetes. JAMA.

[26] Silva PS (2015). Peripheral lesions identified on ultrawide field imaging predict increased risk of diabetic retinopathy progression over 4 years. Ophthalmology.

[27] Marcus DM (2022). Association of predominantly peripheral lesions on ultra-widefield imaging and the risk of diabetic retinopathy worsening over time. JAMA Ophthalmol..

[28] Fan W (2019). Distribution of nonperfusion and neovascularization on ultrawide-field fluorescein angiography in proliferative diabetic retinopathy (RECOVERY study): Report 1. Am. J. Ophthalmol..

[29] Nagasawa T (2019). Accuracy of ultrawide-field fundus ophthalmoscopy-assisted deep learning for detecting treatment-naïve proliferative diabetic retinopathy. Int. Ophthalmol..

[30] Cai S (2021). Deep learning detection of sea fan neovascularization from ultra-widefield color fundus photographs of patients with sickle cell hemoglobinopathy. JAMA Ophthalmol..

[31] Holomcik D (2022). Segmentation of macular neovascularization and leakage in fluorescein angiography images in neovascular age-related macular degeneration using deep learning. Eye.

[32] Li W (2022). A weakly supervised deep learning approach for leakage detection in fluorescein angiography images. Transl. Vis. Sci. Technol..

[33] Lepore D (2020). Convolutional neural network based on fluorescein angiography images for retinopathy of prematurity management. Transl. Vis. Sci. Technol..

[34] Mehta N (2020). Model-to-data approach for deep learning in optical coherence tomography intraretinal fluid segmentation. JAMA Ophthalmol..

