# Supplementary material for: Deep learning for automated detection of neovascular leakage on ultra-widefield fluorescein angiography in diabetic retinopathy
Source: Sci Rep. 2023 Jun 6;13:9165. doi: 10.1038/s41598-023-36327-6 (PMC10244419; doi:10.1038/s41598-023-36327-6)
Supplement: Supplementary file 1 — Supplementary Legends. [file 41598_2023_36327_MOESM1_ESM.docx]

Supplemental Figure 1. Representative images of false negative predictions. In A, there are multifocal areas of neovascularization with relatively faint leakage. In B, small foci of neovascularization are present near the optic disc and inferior to the optic disc. In C, faint areas of neovascularization are present in the inferotemporal macula and directly nasal to the disc. In D, an area of neovascularization is present superonasal to the optic disc. In E, a single focus of neovascularization with faint leakage pattern is present superiorly, along with multifocal areas of late leakage not from neovascularization. In F, a tiny focus of neovascularization is present temporally along a retinal vessel.

Supplemental Figure 2. Representative images of false positive predictions. In A, a chorioretinal scar unrelated to diabetic retinopathy exists inferotemporal to the macula.

multifocal areas of leakage due to vasculopathy are present nasally and superiorly. In B, several areas of retinal vessel staining and late leakage are present. In C, a chorioretinal scar with staining exists in the temporal macula. In D, several areas of retinal vessel staining are present. In E, there is a tiny focus of non-neovascular hyperfluorescence on the optic disk. In F, there are multifocal clusters of microaneurysms nasal to the optic disc.

Supplemental Figure 3. Ten randomly selected images and corresponding saliency maps of true-positive predictions. Examples were selected at random from the pool of true-positive predictions.

Supplemental Figure 4. Ten randomly selected images and corresponding saliency maps of true-negative predictions. Examples were selected at random from the pool of true-negative predictions.

Supplemental Figure 5. Ten randomly selected images and corresponding saliency maps of false-positive predictions. Examples were selected at random from the pool of false-positive predictions.

Supplemental Figure 6. Ten randomly selected images and corresponding saliency maps of false-negative predictions. Examples were selected at random from the pool of false-negative predictions.
