# Supplementary figures and images for: Deep learning for automated detection of neovascular leakage on ultra-widefield fluorescein angiography in diabetic retinopathy
Source: Sci Rep. 2023 Jun 6;13:9165. doi: 10.1038/s41598-023-36327-6 (PMC10244419; doi:10.1038/s41598-023-36327-6)

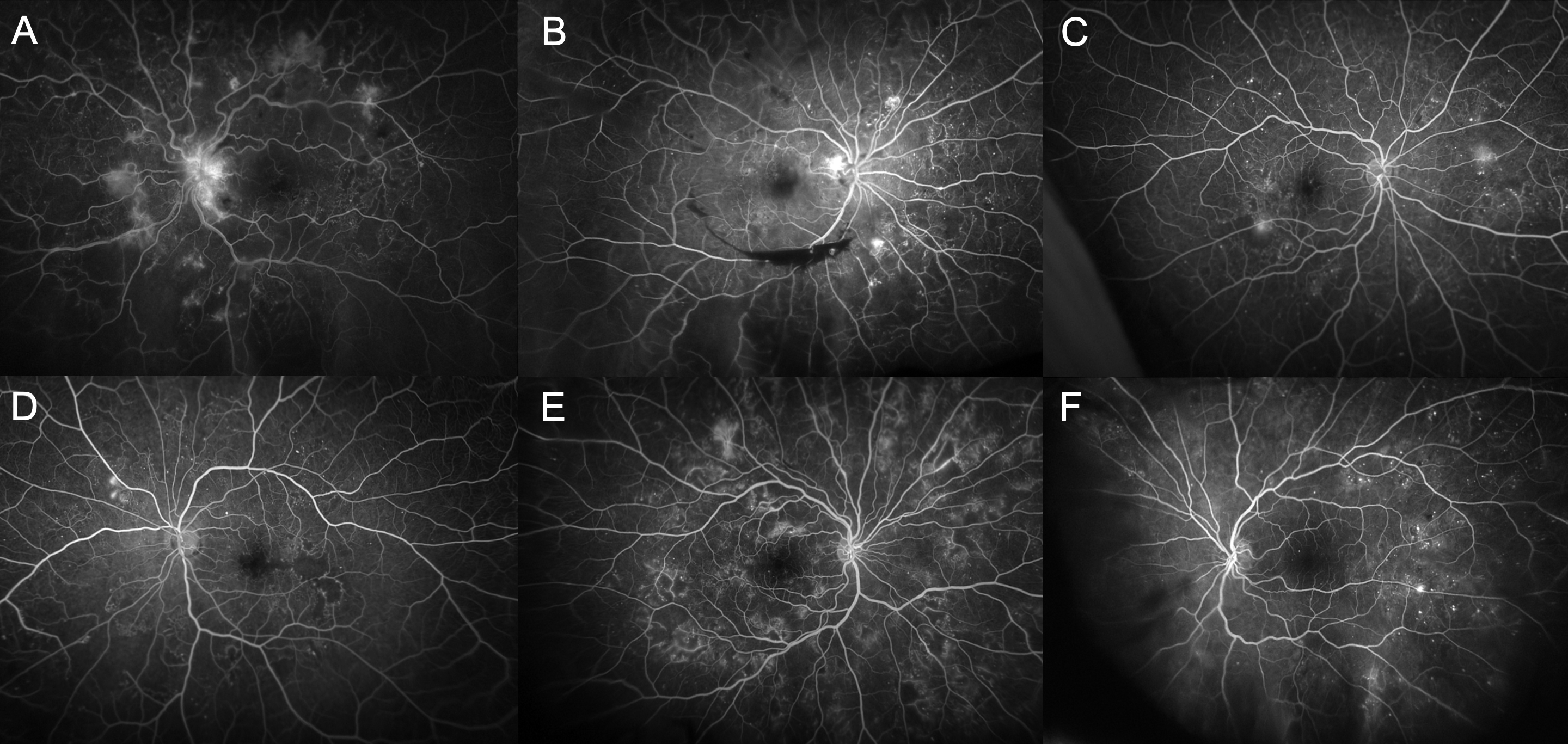

Supplement: Supplementary file 2 — Supplementary Figure S1. [file 41598_2023_36327_MOESM2_ESM.png]

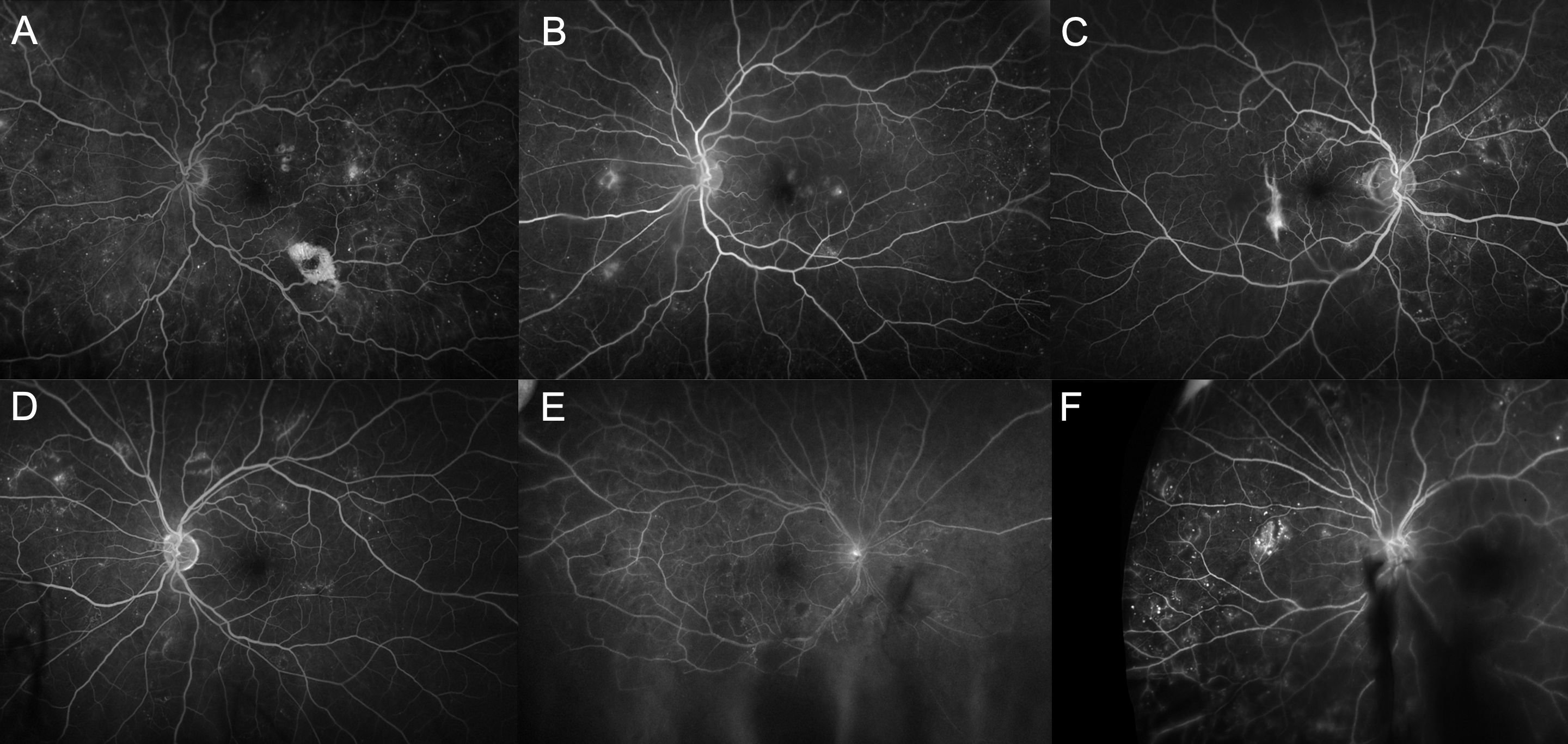

Supplement: Supplementary file 3 — Supplementary Figure S2. [file 41598_2023_36327_MOESM3_ESM.png]

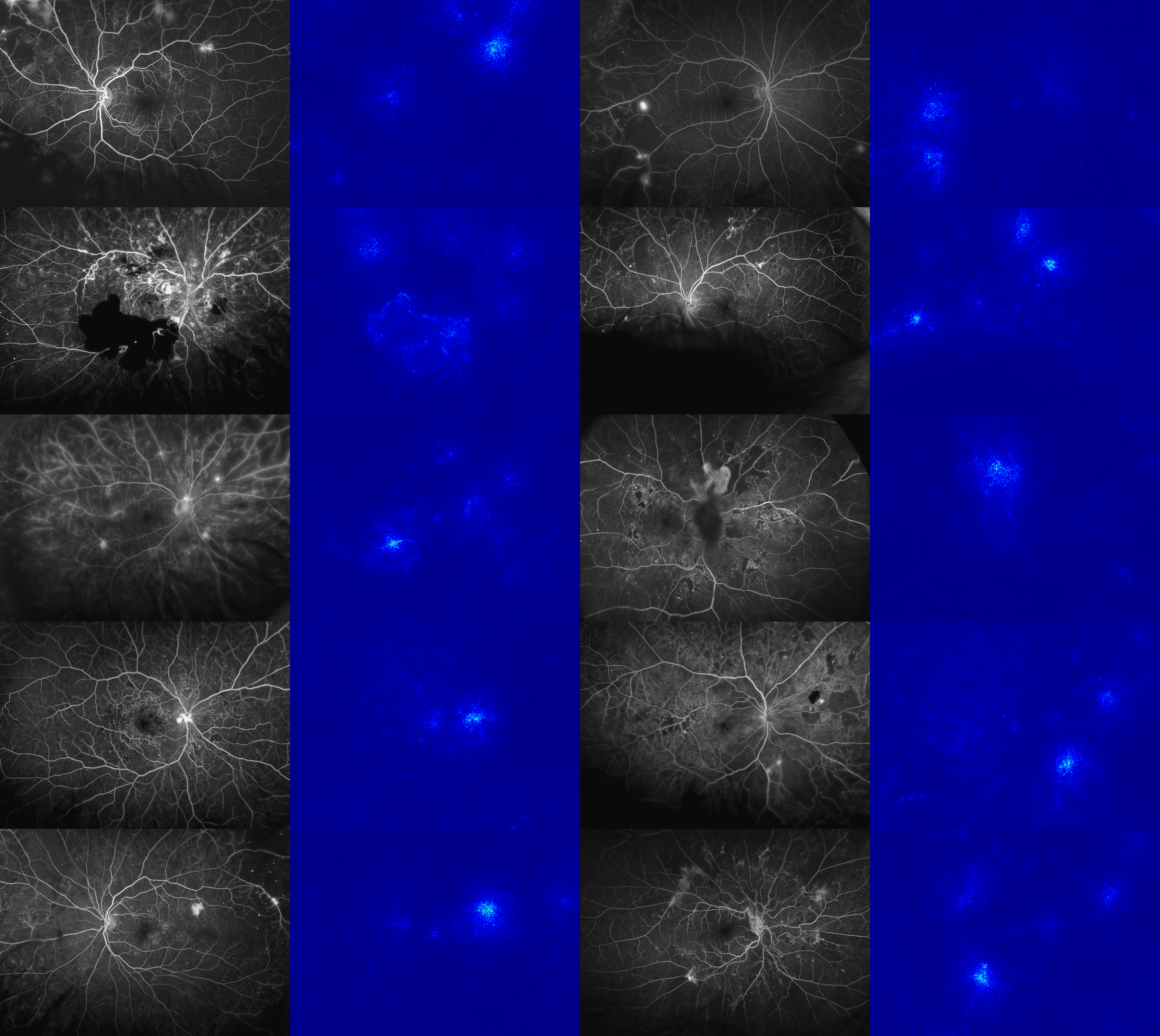

Supplement: Supplementary file 4 — Supplementary Figure S3. [file 41598_2023_36327_MOESM4_ESM.png]

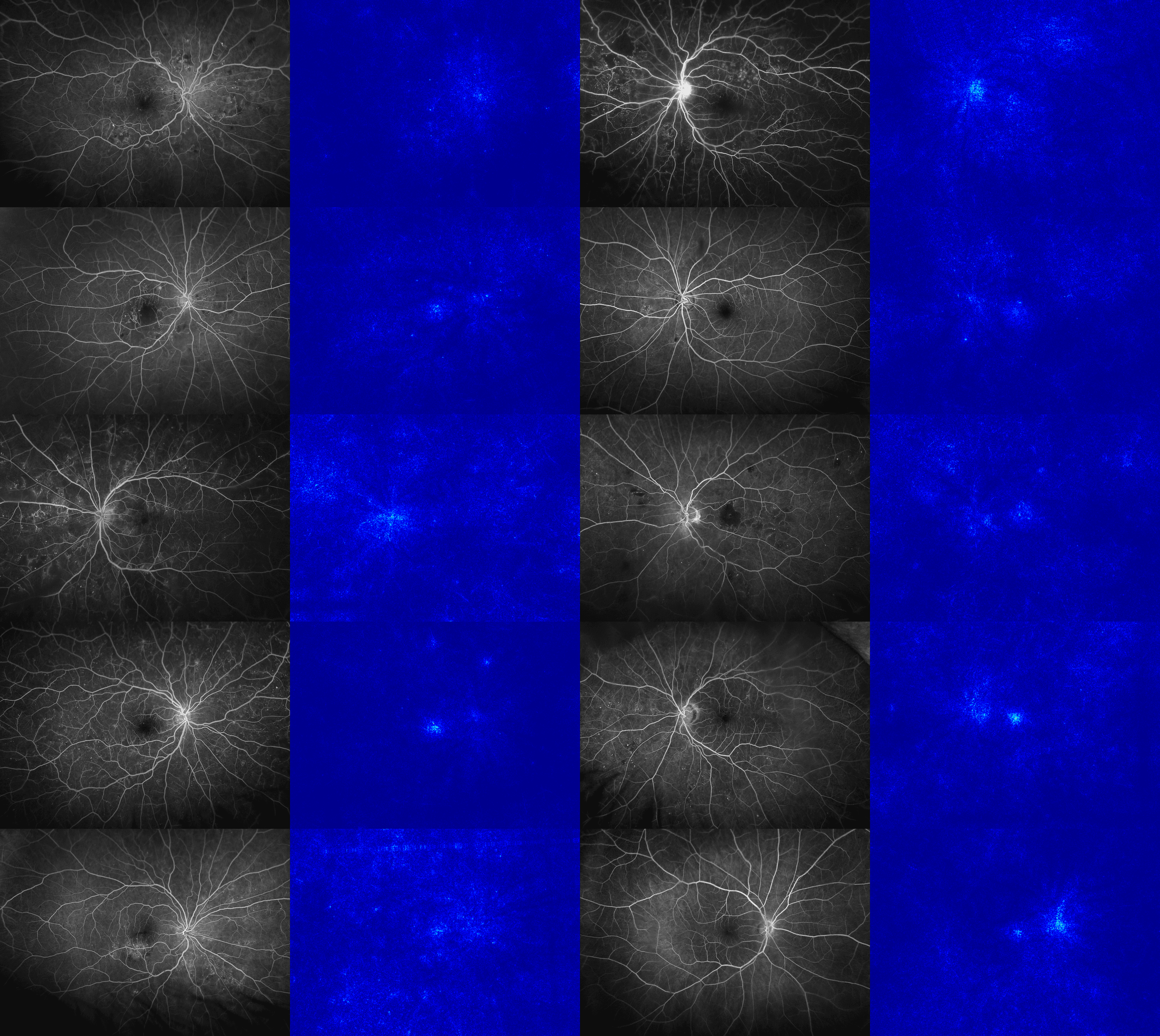

Supplement: Supplementary file 5 — Supplementary Figure S4. [file 41598_2023_36327_MOESM5_ESM.png]

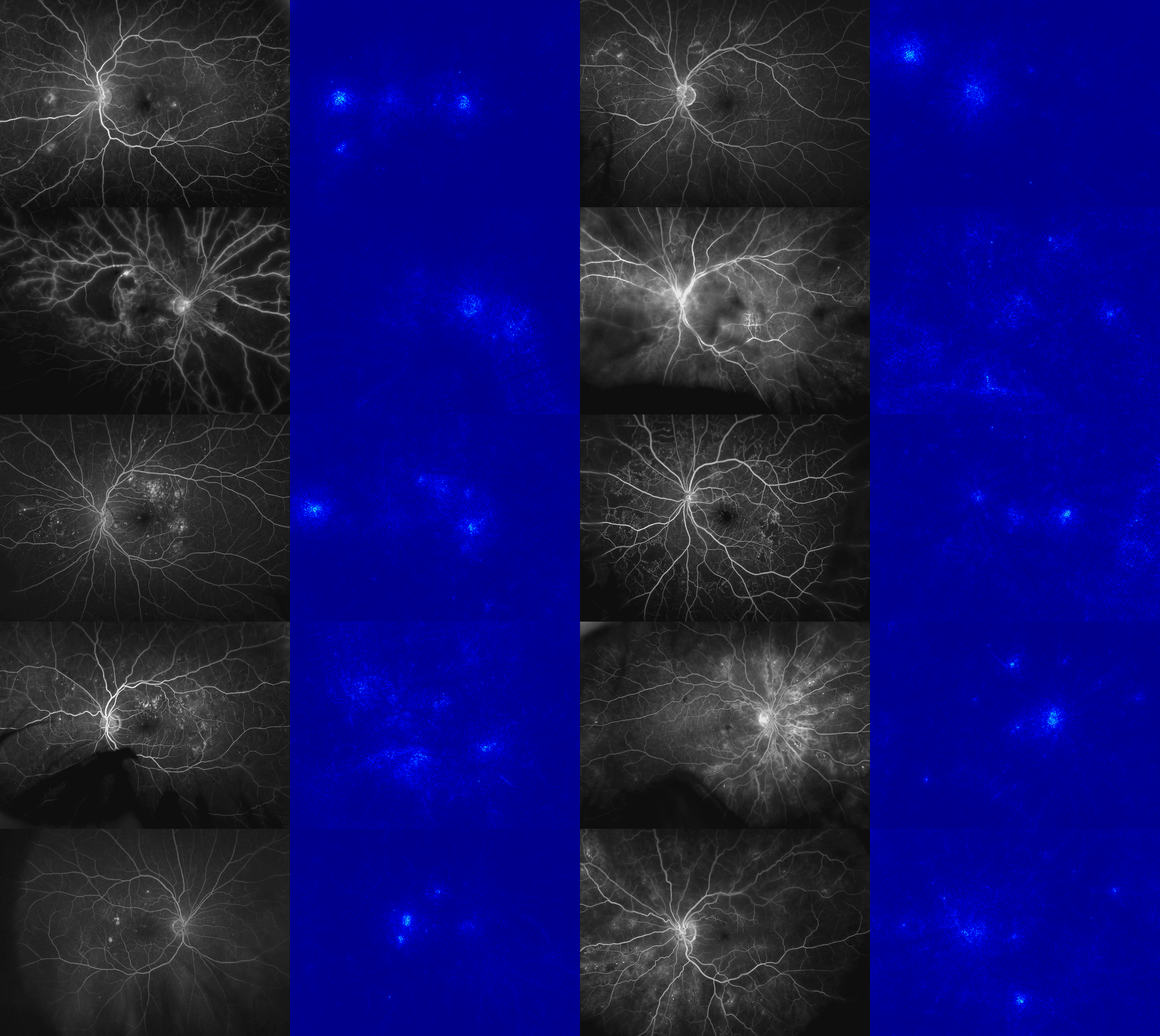

Supplement: Supplementary file 6 — Supplementary Figure S5. [file 41598_2023_36327_MOESM6_ESM.png]

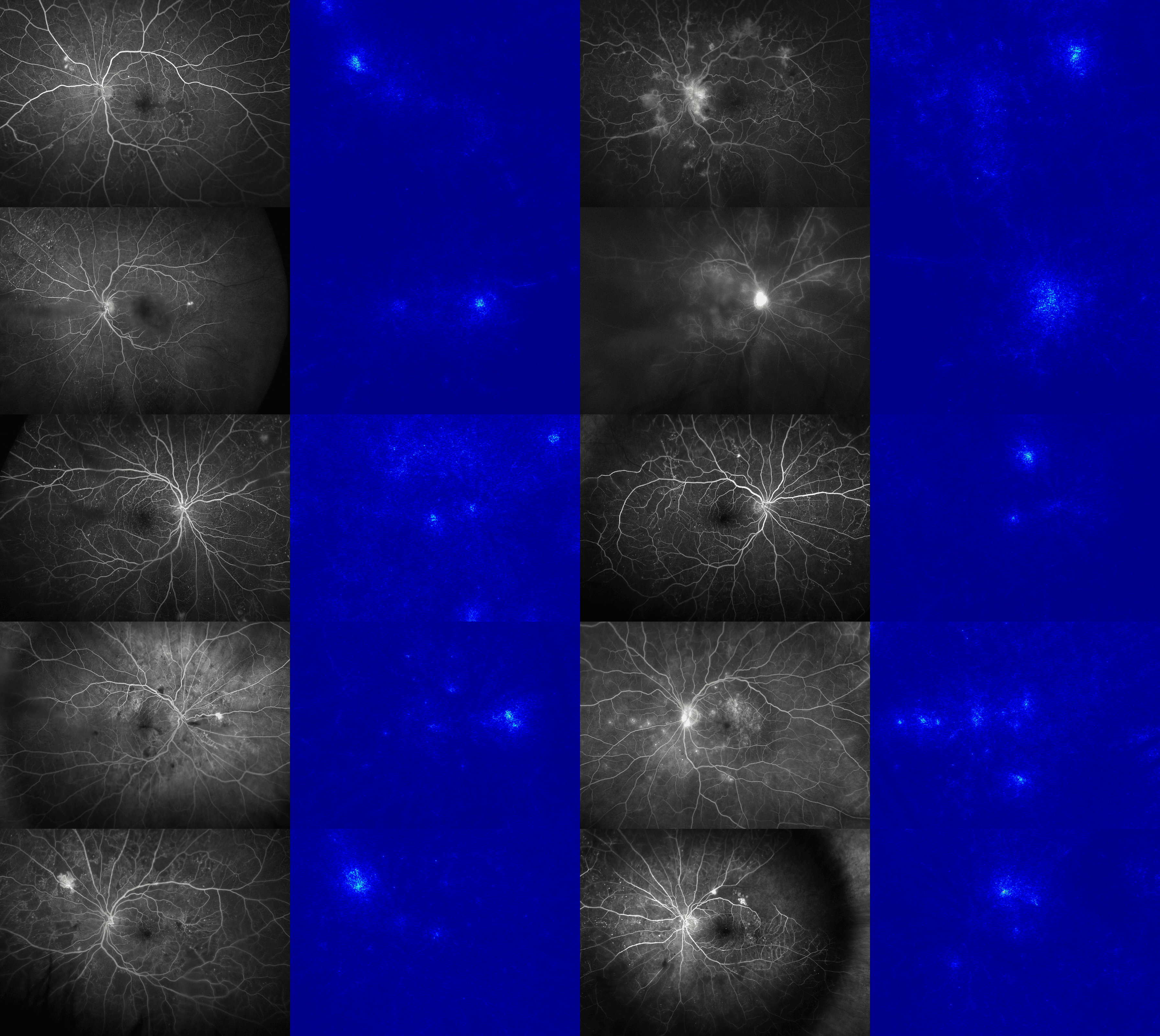

Supplement: Supplementary file 7 — Supplementary Figure S6. [file 41598_2023_36327_MOESM7_ESM.png]
